# Robotic-assisted systems for the safe and reliable treatment of femoral neck fractures: retrospective cohort study

**DOI:** 10.1186/s13018-023-04070-3

**Published:** 2023-08-28

**Authors:** Xiaofei Wang, Yaxin Zhang, Linbing Lou, Lei Xu, Wenyong Fei, Jihang Dai, Jingcheng Wang

**Affiliations:** 1 Dalian Medical University, Dalian, 116044 China; 2 Department of Orthopedics, Northern Jiangsu People’s Hospital, Clinical Medical College, Yangzhou University, Yangzhou, 225001 China

**Keywords:** Minimally invasive, Orthopedic robot, Femoral neck fracture, Internal fixation

## Abstract

**Background:**

Robots are being used in a wide range of surgical procedures. However, in clinical practice, the efficacy of orthopedic robotic-assisted treatment of femoral neck fractures is still poorly reported, particularly in terms of screw placement accuracy, femoral neck fracture healing rates and postoperative functional recovery. Moreover, there is a lack of comparative analysis between robot-assisted surgery and traditional surgical approaches.

**Purpose:**

The purpose of this study was to compare the clinical outcomes of patients with femoral neck fractures treated with TiRobot-assisted hollow screw fixation with those of patients with femoral neck fractures treated with traditional surgical approaches.

**Methods:**

This study included 112 patients with femoral neck fracture who were treated from March 2017 to October 2021 with percutaneous hollow screw internal fixation. These included 56 cases in the TiRobot-assisted surgery group and 56 cases in the standard surgery group. After at least 1 year of follow-up, the treatment outcomes of the two groups were compared, including the amount of intraoperative bleeding, the duration of intraoperative fluoroscopy, the number of guide pin positioning adjustments, the length of hospital stay, the accuracy rate of screw placement, the final Harris Hip Score, the fracture healing rate, and the rate of femoral head necrosis. Statistical analysis software was used to process and analyze the result.

**Results:**

The TiRobot-assisted group had a statistically significant improvement over the control group in terms of intraoperative bleeding, the duration of intraoperative fluoroscopy, the number of guide pin positioning adjustments, length of hospital stay, accuracy of screw placement and incidence of femoral head necrosis (*P* < 0.05). There was no statistically significant difference in time to surgery, final Harris hip score and fracture healing rate (*P* > 0.05).

**Conclusion:**

This study shows that TiRobot-assisted surgery has the advantages of short hospital stay, high safety, minimally invasive, high success rate of nail placement, and can reduce the amount of intraoperative radiation and the incidence of femoral head necrosis, thus achieving satisfactory clinical outcomes, and is worthy of clinical promotion.

## Introduction

According to relevant reports, there is currently a gradual increase in the number of hip fracture patients worldwide. It is estimated that 4.5 million people are disabled by hip fracture each year, and it is expected that the number of people disabled by hip fracture will increase to 21 million in the next 40 years [1, 2]. The increased incidence of hip fractures also leads to higher healthcare costs [3].

Among patients with hip fractures, the prevalence in older patients increases progressively with increasing life expectancy [4]. However, younger patients usually suffer from high-energy trauma, such as falls from heights or high-speed traffic accidents [5]. Femoral neck fracture is one of the most common hip fractures, and despite surgical intervention in most patients, the number of patients requiring reoperation continues to grow steadily [6, 7].

The treatment of femoral neck fractures is currently based on arthroplasty and repositioned internal fixation [8].However, complications such as non-union of the fracture and ischemic necrosis of the femoral head occur more commonly after common internal fixation, which will seriously affect the functional recovery of patients after surgery and lead to secondary surgery. How to reduce the complications of the femoral neck fracture internal fixation have always been a problem that plagues clinicians and the research of many scholars [9]. Some researchers have suggested that an adequate perioperative treatment modality is essential to reduce mortality and avoid complications [10].

Currently, artificial intelligence (AI) is being widely used in orthopedic surgery, especially to guide the increasing application of minimally invasive internal fixation, where, by integrating patient imaging data, robots can establish more rational treatment patterns, simplify clinical operations, and improve the accuracy of surgical treatment [11–13]. Its use to assist hollow screw internal fixation for femoral neck fractures has been reported, and the results showed advantages in terms of minimally invasive surgery and intraoperative adjustment of the number of internal fixations [14].

The TiRobot (TINAVI Medical Technologies, Beijing, China), which is applied in this study, is a general-purpose orthopedic navigation robot that uses intelligent algorithms to calculate the trajectory of the guided screws, which can make orthopedic surgery more precise. Robot-assisted orthopedic surgery is an eye-brain-hand coordination process like conventional surgery, and there have been reports related to TiRobot for femoral neck fractures [15, 16]. Although the effect of treating femoral neck fractures with the help of robotics in clinical practical application is still controversial, some researchers have explained that robots have some advantages in treating femoral neck fractures [17].

The aim of this retrospective cohort study is to compare and analyze the differences in clinical efficacy between the two groups of patients with different surgical modalities, which mainly includes comparing the safety and reliability of the surgery. In addition, the length of hospital stay, incidence of femoral head necrosis, and hip function after different surgical modalities were also included in our comparison. These can provide some references for the therapeutic efficacy of robotic-assisted system applied to femoral neck fracture.

## Methods

### Patient information

One hundred and twelve patients with femoral neck fractures treated with percutaneous hollow screw internal fixation from March 2017 to October 2021 were selected, including 62 males (about 55%) and 50 females (about 45%).The age range was 16—81 years (mean age 61.3 ± 7.7 years for both sexes). According to the Garden classification, there were 12 type I, 41 type II, 46 type III and 13 type IV fractures.

The general data of the patients in both groups, gender, age, BMI (body mass index), and fracture Garden typing were statistically compared, and it was found that there was no statistically significant difference between the groups (*P* > 0.05) (Table 1).

**Table 1:** Comparison of the general information of the two groups of patients (mean ± standard deviation)

Indicators	TiRobot	Control	*P*
Number of persons (cases)	56	56	–
Gender (cases)			
Male	32	30	0.352
Female	34	36	
Age (years,$\overline{x}$ ± s)	59.5 ± 8.7	60.1 ± 8.2	0.450
BMI(kg/m2, $\overline{x}$ ± s)	23.1 ± 2.4	22.8 ± 1.9	0.196
Garden typing			
Type I	5	7	0.904
Type II	22	19	
Type III	22	24	
Type IV	7	6	

## Inclusion and exclusion criteria

- **Inclusion criteria:** 1: all types of femoral neck fractures aged < 60 years, and Garden type I and II III femoral neck fractures aged > 60 years; 2: experienced a follow-up period of 1 year; 3: the patient is not injured or hospitalized for more than 14 days [18].
- **Exclusion criteria:** 1: garden Fracture Classification Type IV at > 60 years of age; 2: multiple femur fractures; 3: patients with severe osteoporosis; 4: patients with other difficult-to-tolerate combined diseases.

### Surgical equipment

The surgery was performed by the TiRobot system, the third generation of the TIANJI orthopedic robot. The system equipment consisted of the robot, surgical planning, spatial alignment components, robot control software, an optical tracking system, a master control station, and supporting tools. The C-arm X-ray system (Siemens, Germany), orthopedic traction bed, and other equipment were used together in the operation.

### Surgical procedure (TiRobot assisted)

The surgeries were performed by the same treatment team with many years of experience in fracture of the femoral neck, and the lead surgeon was a highly qualified physician trained in robotic techniques. All patients underwent X-rays and 3D imaging prior to surgery, and the team planned the number and placement of screws.

All patients were put under general anesthesia, the patients were lying flat on the traction bed, the affected limbs were tractioned, and the fracture was reset by manipulation such as internal retraction, abduction, internal rotation, external rotation, hip flexion, and knee flexion. Fluoroscopy with a "C" arm X-ray machine showed that the fracture was close to anatomical restoration and was compared with the contralateral intact side. Indeed, correct traction followed by maintenance of anatomical reduction of the fracture is a critical prerequisite for a successful surgical procedure, which is dependent on the surgeon's manipulative technique rather than robot-assisted reduction.

After observing that the anterior tilt angle, neck stem angle, and femoral distance are relatively good. Perform preoperative preparatory operations such as sterilization and towelling of the lower limbs.

**Fig. 1:**
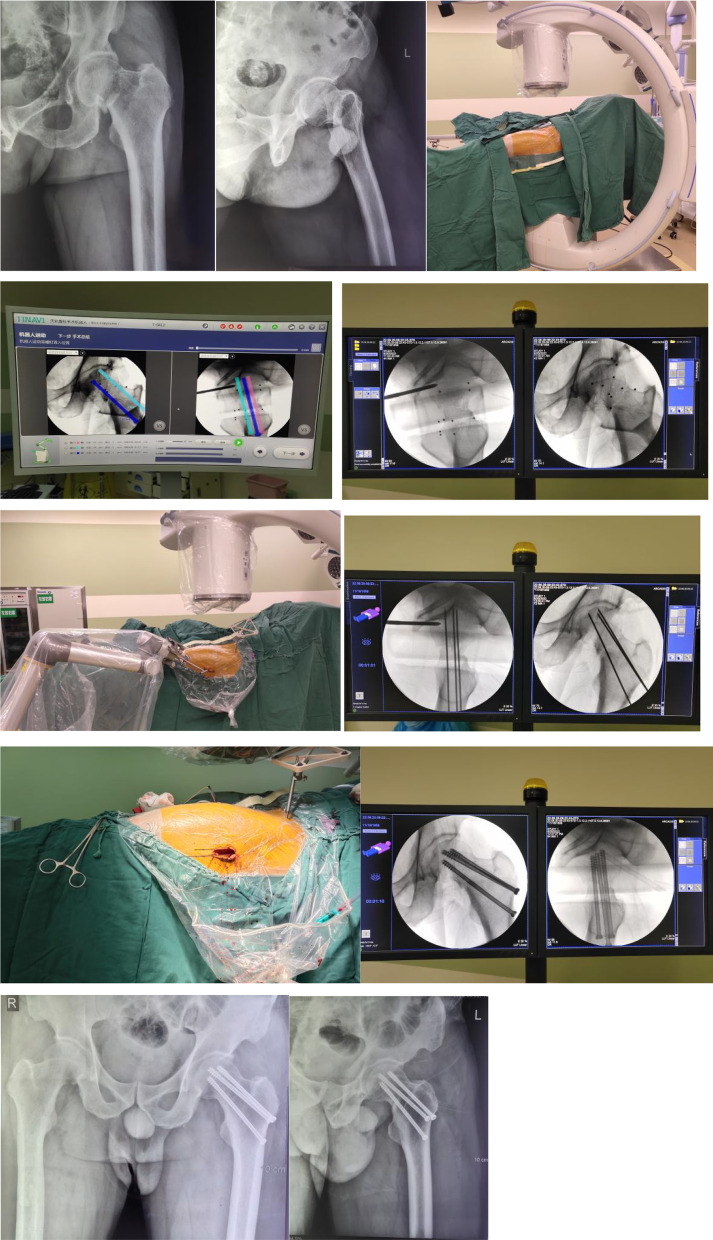
Patient 1, male, 56 years old, left femoral neck fracture (**a**, **b**) X-ray examination shows: left femoral neck fracture, Garden type III. **c** After traction on the affected limb and satisfactory alignment of the femoral neck fracture, routine lower limb disinfection was performed. **d**, **e** Computerized planning of the femoral neck hollow nail placement path, length and diameter by the robot. **f–h** Use the surgical robot to position the guide cannula in the desired position, then drive three parallel guide pins in turn, fluoroscopy the guide pin position, direction, and length as needed. **i** The hollow drill bit is drilled through the lateral femoral bone cortex with the guide pins, the 7.3 mm diameter hollow screw is selected for placement with the guide pins, and the screw is tightened with gradual pressure. **j**, **k** Postoperative X-ray fluoroscopy showed satisfactory fracture repositioning and hollow screw position

**Fig. 2:**
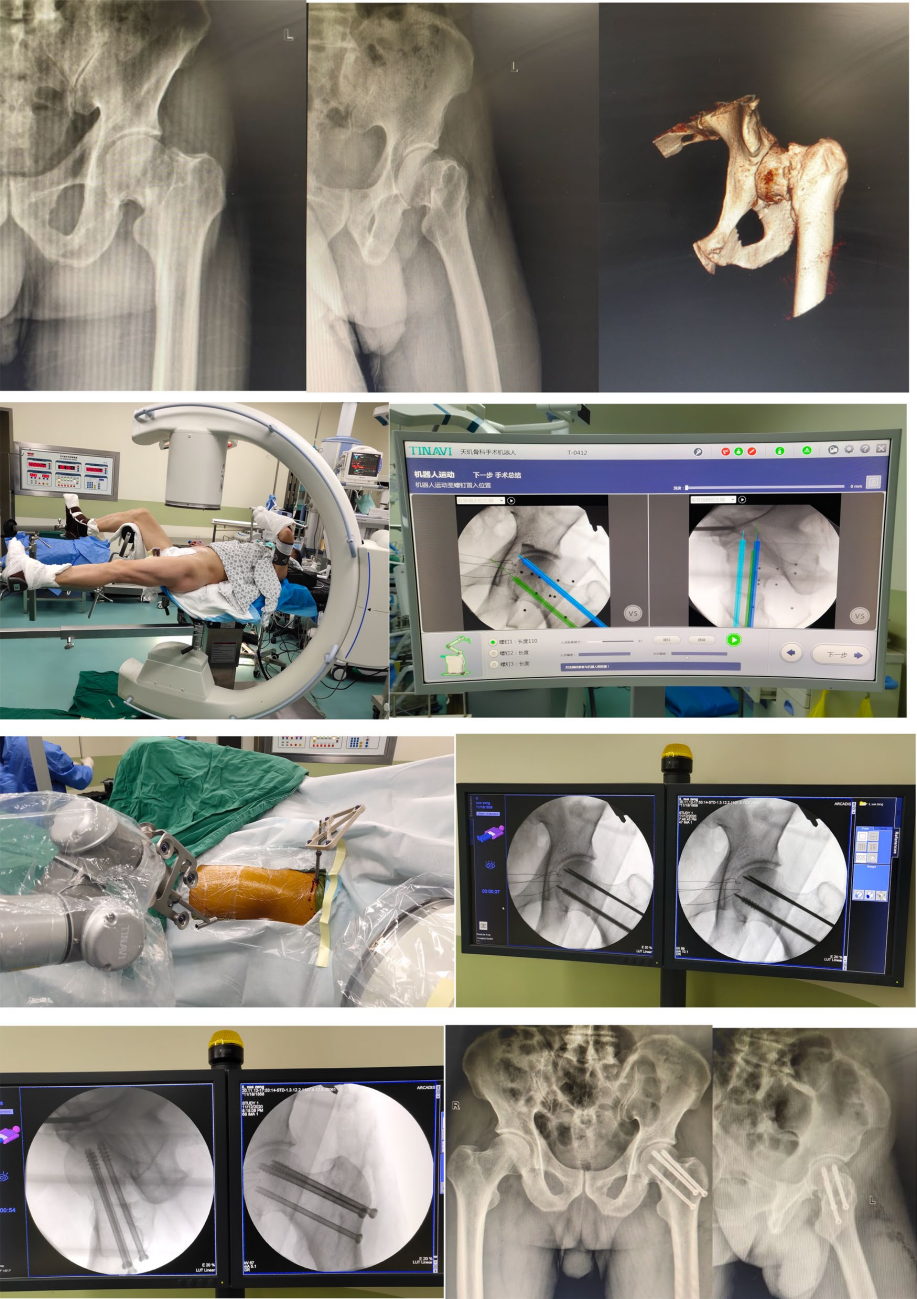
Patient 2, male, 61 years old, **a**, **b** X-ray showed: left femoral neck fracture, Garden type III. **c **The affected limb was first placed in traction until the alignment of the femoral neck fracture was satisfactory, and then routine lower limb disinfection was performed. **d**, **e** The robot's computer plans the path, length, and diameter of the femoral neck hollow nail placement. **f**–**h** Then, the surgical robot is run to position the guide cannula in the planned position, and three parallel guide pins are drilled in sequence, with the position, direction, and length of the guide pins again fluoroscopically appropriate. **i** Finally, the hollow drill bit is drilled through the lateral femoral bone cortex with the guide pins, and the 7.3-mm diameter hollow screw is selected for placement with the guide pins, and the screw is tightened with gradual pressure. **j**, **k** Postoperative X-ray fluoroscopy showed satisfactory fracture repositioning and hollow screw position

An optical tracer was placed on the anterior superior iliac spine, the robot arm and the positioning scale were placed in the appropriate position, and the standard frontal and lateral images of the femoral neck were acquired by the C-arm X-ray machine. After running the surgical robot, the guide cannula was positioned in the planning position, and 3–4 parallel guide pins were driven in sequence, The position, direction, and length of the guide pins were again fluoroscopically appropriate. After measuring the depth of the bone tract, a hollow drill was used to drill through the lateral femoral cortex with the guide pins, and a 7.3-mm diameter hollow screw was selected for placement with the guide pins, and the screw was tightened with gradual pressure. Finally, the fluoroscopic examination of fracture repositioning and hollow screw position was satisfactory (Figs. 1, 2).

### Surgical procedure in the standard group

After the broken end of the fracture is successfully repositioned, routine disinfection is performed. Firstly, under C-arm fluoroscopy, the insertion position and angle of the guide pin need to be adjusted repeatedly. Subsequently, the guide pin is gradually advanced using the guide pin under the best frontal and lateral images until the cartilage level of the femoral head is reached. Finally, 3–4 7.3 mm diameter hollow screws were selected for placement with the guide pins, and the screws were tightened with gradual pressure. After fluoroscopy showed that the screws were well positioned, the guide pins were removed and the skin was sutured layer by layer.

### Postoperative treatment and efficacy evaluation index

Patients in both groups were treated in the same way after surgery. The patients were advised to avoid extreme external rotation, abduction, and adduction of the affected hip for 6 weeks. The hip X-ray, including the orthogonal and lateral hip X-rays of the affected hip, was reviewed on the second postoperative day. The healing of the femoral neck fracture was reviewed and observed at 1 month, 3 months, 6 months, and 1 year after surgery.

After at least 1 year of follow-up, the two groups were compared in terms of treatment outcome, which included intraoperative bleeding, intraoperative fluoroscopy time, number of guide pin position adjustments (number of adjustments made during guide pin fixation of fractures. It is necessary to adjust the guide pins according to the position of the guide pins in relation to the fracture fixation as observed by X-ray fluoroscopy), length of hospital stay, accuracy of screw placement, final Harris hip score, fracture healing rate and incidence of femoral head necrosis. Finally, the results were processed and analyzed using statistical analysis software.

### Statistical analysis

The data were analyzed using SPSS 25.0 statistical software. The mean ± standard deviation ($\overline{x}$ ± *s*) was used to express the normal distribution of the measures, and the median (lower quartile, upper quartile) was used for the non-normal distribution. Comparisons between groups were made using the independent samples t-test. The chi-square test was applied to the statistical data.* P* < 0.05 is considered statistically significant.

## Results

All patients completed the surgery successfully, and all chose the lateral femoral incision without complications such as neurovascular injury and incisional infection.

The results showed that the TiRobot-assisted group had better intraoperative bleeding, intraoperative fluoroscopy time, number of guide pin position adjustments, length of stay and incidence of femoral head necrosis than the control group, which was statistically significant (*P* < 0.05). There was no statistically significant difference in the time to surgery, final Harris hip score and fracture healing rate (*P* > 0.05).

Postoperative orthogonal and lateral radiographs of the hip joint were collected from patients in the TiRobot and control groups, and these images were imported into computer software and the screw placement accuracy was calculated. The software calculated the deviation angle of the screw from the neck-shaft angle and anterior tilt angle placement, respectively, according to the corresponding algorithms. The results of the analysis showed that the difference between the TiRobot group and the control group was statistically significant (*P* < 0.05) in the deviation angles of the neck-shaft angle and anterior tilt angles (Table 2).

**Table 2:** Comparison of general results between the two groups (mean ± standard deviation)

Results	TiRobot	Control group	*P*
Intraoperative bleeding(ml)	11.73 ± 2.88	70.63 ± 16.32	*P* < 0.001
Intraoperative fluoroscopy time (s)	29.16 ± 2.41	56.68 ± 9.30	*P* < 0.001
Number of needle position adjustments (times)	6.05 ± 1.67	15.88 ± 4.82	*P* < 0.001
Length of hospitalization (Number of days)	8.82 ± 1.83	11.04 ± 2.02	*P* < 0.001
Time to surgery(min)	73.29 ± 10.62	73.46 ± 11.31	0.932
Final Harris hip score (score)	89.48 ± 4.63	89.30 ± 4.32	0.833
Screw deviation angle from neck-shaft angle(angle)	3.77 ± 1.19	5.71 ± 1.47	*P* < 0.001
Screw deviation angle from anterior tilt angle(angle)	6.70 ± 1.63	9.40 ± 2.10	*P* < 0.001
Fracture healing rate (%)	96.43(54/56)	92.86(52/56)	0.675
Femoral head necrosis (%)	3.57(2/56)	19.62(11/56)	*P* < 0.05

## Discussion

As healthcare technology is growing exponentially and AI technologies are introduced in healthcare, one of the most intriguing technologies is currently robot-assisted surgical operations. The potential benefits of robotic surgery include improved surgical workflow, increased efficacy, and reduced operative time. Parsley argues that we are entering a brave new world with the era of artificial intelligence and orthopedic robotics [19]. The avenues we can see in the application of AI whether it is for survival, cost prediction, assisting with image diagnosis, clinical decision support, or even implant design and improvement are vast and varied [11].

### Problems associated with conventional surgery

Femoral neck fracture is a common hip fracture, and anatomical repositioning and effective fixation are the keys to a good outcome of hip preservation therapy after femoral neck fracture, and satisfactory results can be achieved in about 59% of patients by closed repositioning [1, 20]. The femoral neck is in a special position, with the femur located within the joint capsule, and the surface of the femoral neck is not covered by muscles, blood vessels, or other soft tissues, so the blood supply is poor, and after the fracture, the basic blood supply is destroyed, which makes healing difficult and can easily cause ischemic necrosis of the femoral head. This anatomical structure can easily cause the hollow nail to penetrate the bone cortex during surgery, leading to a series of postoperative complications and ultimately to surgical failure [21, 22]. When operating in the traditional surgical way, there is no way to judge the position precisely, which can easily cause deviation. And it is more difficult to control when adjusting the position of the screw placement, which has the possibility of causing medical injuries. At the same time, unsuccessful surgical operations can lead to longer operating times and increase the surgeon's exposure to radiation during the operation.

There is variability among orthopedic surgeons in terms of optimal treatment and variations in treatment trends with regard to the choice of surgical approach for patients with femoral neck fractures [23]. When choosing to treat displaced femoral neck fracture patients younger than 60 years of age, relevant scholars believe that primary total hip arthroplasty can be performed due to the presence of risk factors and the fact that osteotomies are associated with a high risk of complications (ischemic necrosis of the femoral head, bone non-union) [24]. However, some other researchers have argued that anatomical reduction and stable internal fixation are recommended for patients under 70 years of age, regardless of the degree of displacement of the fracture, except for those with severe disease and risk of over-operation [25].

In this study, we believe that treatment should be individualized according to the patient's condition. Firstly, we excluded patients who were older than 65 years and had more severely displaced fractures. However, there were individual patients who were older but had non-displaced fractures and were in relatively poor general health and could not tolerate arthroplasty. We chose the minimally invasive TiRobot-assisted surgery to reduce pain and improve the quality of life of the patients, and the results were favorable.

### Advantages of TiRobot system assisted surgical operation

The TiRobot system surgery, with the help of artificial intelligence, significantly improves the accuracy of placing hollow screws, significantly reduces operation time, and significantly reduces the number of fluoroscopies. The TiRobot robotic system has the only registration license from the China Food and Drug Administration and has been certified for use in orthopedic surgery. The system is one of the most advanced multifunctional orthopedic surgical robotic navigation systems in the world, with the advantages of simple operation, precise positioning, minimal invasiveness, and minimal radiation exposure, representing a combination of stereotactic technology and automated manipulators. Several related authors have reported their experience and the feasibility of its clinical application in orthopedics [26–28]. This study confirms the advantages of the TiRobot system in the treatment of femoral neck fractures, provided that the fracture is in good position after traction and repositioning, so good preoperative traction and repositioning is a prerequisite for the success of the procedure.

The rapid and precise placement of the guide pin during the surgical operation reduces the bone damage caused by repeated adjustment of the guide pin to achieve a satisfactory internal fixation position in traditional surgery. Especially in middle-aged and elderly patients with osteoporosis (non-severe), the accuracy of internal fixation placement is improved by avoiding the repeated penetration of the guide pin that would cause bone pitting in the access area. In addition, the TiRobot system reduces the length of the incision and intraoperative bleeding in a precise mode of operation, speeding up the patient's postoperative recovery and conforming more to the concept of rapid rehabilitation, which also shortens the patient's hospital stay. Relevant scholars believe that hip fracture patients have decreased muscle mass and even loss of physical function [29]. Therefore, rapid postoperative rehabilitation of patients is particularly important.

The TiRobot system offers the advantage of a more minimally invasive procedure than traditional surgery and reduces the incidence of femoral head necrosis. Relatively speaking, there is no significant difference in the healing rate of femoral neck fractures and functional recovery of the hip joint. However, further follow-up observations are required for patients with possible femoral head necrosis after 2–3 years.

### Limitations of robot-assisted systems

Firstly, the TiRobot operation still requires good traction and fracture reduction prior to surgery in order to maintain the fracture position of the femoral neck to its advantage. In addition, the operation of the orthopedic surgery robot needs to be performed by experienced surgeons, especially for the path planning of screws during surgery, which has a certain learning curve. And, the high cost of surgical robotic machines ($2 million) is one of the factors hospitals need to consider. Finally, orthopedic surgical robots are not fully available in primary hospitals, and patients with traumatic fractures may not be able to choose a medical unit for timely consultation.

### Application prospect and outlook

As orthopedic robot-assisted surgery becomes more widely used in clinical practice, the role of the surgeon will also change, gradually evolving from the performer of surgery to the designer of surgery. Therefore, the surgeon's experience and technique are particularly important, and a detailed surgical operation plan needs to be developed. In clinical surgery applications, surgical robotic navigation systems are tools of great significance, promoting the development of orthopedic surgery and even medicine as a whole. It is believed that with the progress and development of medicine, surgical robots will be popularized in other fields of surgery, and the surgical navigation system will be improved even more, bringing benefits to more patients.

## Conclusion

This study shows that TiRobot-assisted surgery has the advantages of short hospital stay, high safety, minimally invasive, high success rate of nail placement, and can reduce the amount of intraoperative radiation and the incidence of femoral head necrosis, thus achieving satisfactory clinical outcomes, and is worthy of clinical promotion.

## Data Availability

Not applicable.
